# Supplementary material for: Influence of Secondary Metabolites According to Maturation of Perilla (Perilla frutescens) on Respiratory Protective Effect in Fine Particulate Matter (PM2.5)-Induced Human Nasal Cell
Source: Int J Mol Sci. 2024 Nov 12;25(22):12119. doi: 10.3390/ijms252212119 (PMC11594022; doi:10.3390/ijms252212119)
Supplement: Supplementary file 1 [file ijms-25-12119-s001.zip › ijms-3213257-supplementary.pdf]

Supplementary Data S1. Distribution of extraction yield [EY], antioxidant compounds (TPC, TFC), antioxidant activities (ABTS and DPPH radical scavenging activity), individual phenolic compounds (caffeic acid [CA], rosmarinic acid [RA], Luteolin [LT], Apigenin [APG]), policosanol (Hexacosanol [HC], Octacosanol [OC], Triacosanol [TC] in perilla seeds and flowers, depending on varieties and growth periods.

Supplementary Data S2. Distribution of extraction yield [EY], antioxidant compounds (TPC, TFC), antioxidant activities (ABTS and DPPH radical scavenging activity) in perilla seeds and flowers, based on varieties and growth periods.

Supplementary Data S3. Distribution of individual phenolic compounds (caffeic acid [CA], rosmarinic acid [RA], Luteolin [LT], Apigenin [APG]) in perilla seeds and flowers, based on varieties and growth periods.

Supplementary Data S4. Distribution of individual policosanol (Hexacosanol [HC], Octacosanol [OC], Triacosanol [TC] in perilla seeds and flowers, based on varieties and growth periods.

Supplementary Data S5. Changes in cell viability of human nasal epithelial cells (RPMI2650) depending on the concentration of PM2.5 stimulation (0, 50, 100, 200, 400 μg/mL).

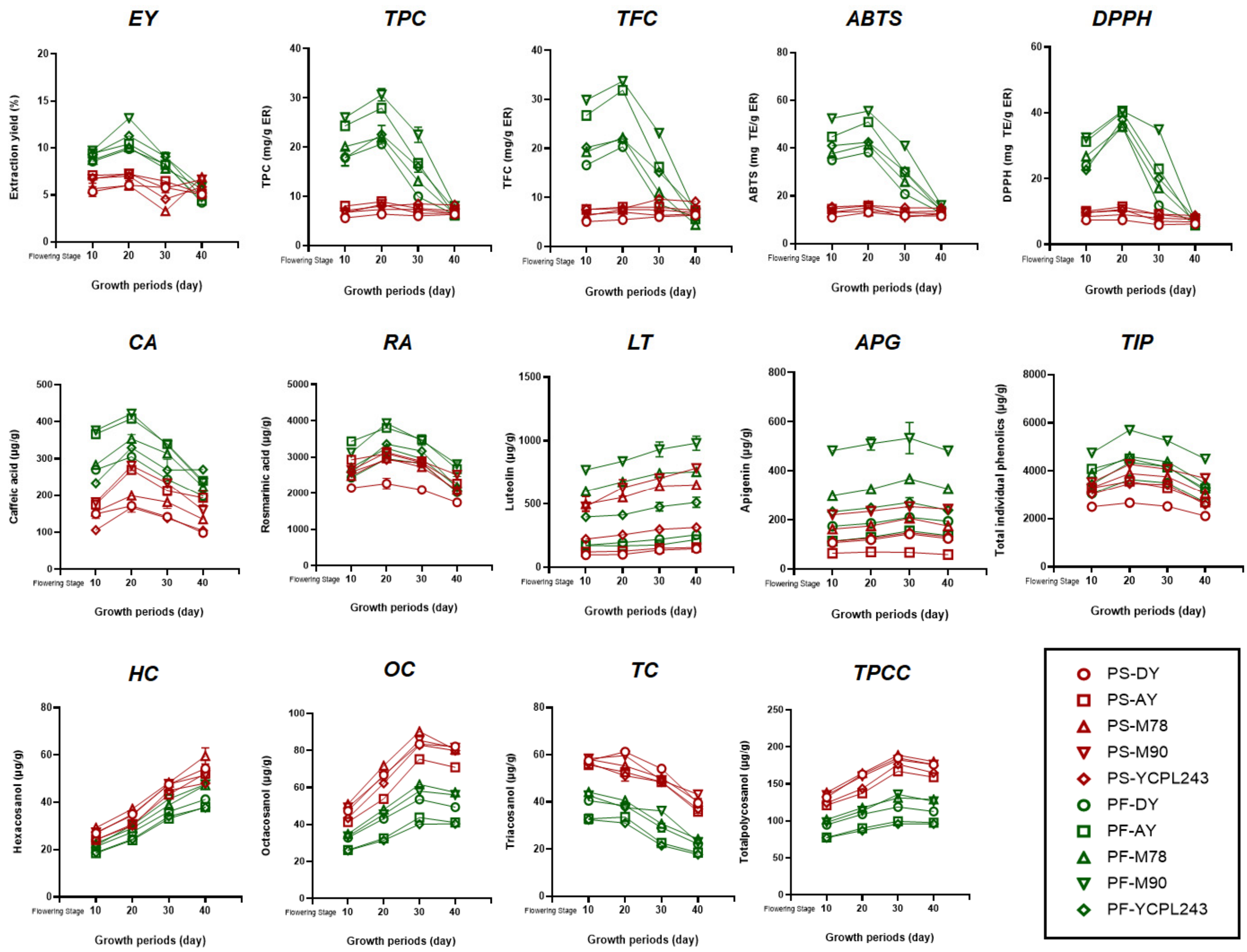

Supplementary Data S1.

Supplementary Data S2.

Part	Varieties		Flowering Stage	Growth Periods	Harvest Stage	Extraction Yeild(%)	Antioxidant component		Antioxidant activity	
							TPC (mg GAE/g ER)	TFC (mg CE/g ER)	ABTS (mg TE/g ER)	DPPH (mg TE/g ER)
Seed	Cultivars	Dayu	9.5	Day 10	9.15	5.37	5.62±0.17	5.10±0.08	11.03±0.43	7.43±0.11
				Day 20	9.25	6.05	6.39±0.22	5.49±0.12	13.16±0.81	7.42±0.57
				Day 30	10.50	5.79	6.02±0.20	6.10±0.20	11.75±0.79	5.99±0.43
				Day 40	10.15	5.08	6.34±0.27	6.41±0.19	11.59±0.72	6.23±0.42
		Anyu	8.25	Day 10	9.40	7.09	8.02±0.16	7.56±0.13	14.55±0.29	10.08±0.13
				Day 20	9.14	7.23	8.90±0.31	8.05±0.02	15.91±0.51	11.41±0.27
				Day 30	9.24	6.47	7.54±0.21	8.03±0.22	12.91±0.97	9.08±0.58
				Day 40	10.40	5.14	7.11±0.32	7.37±0.16	12.54±1.20	7.81±0.49
	Elite Line	Milyang 78	9.2	Day 10	9.12	5.67	6.79±0.18	6.31±0.16	13.16±1.11	8.45±0.11
				Day 20	9.22	6.02	7.34±0.34	7.54±0.19	13.56±1.03	9.07±0.40
				Day 30	10.20	3.29	7.31±0.23	7.50±0.10	13.30±1.25	8.03±0.27
				Day 40	10.12	6.90	6.58±0.18	6.91±0.16	13.74±0.75	7.62±0.22
		Milyang 90	8.3	Day 10	9.90	6.72	6.63±0.23	6.54±0.19	13.18±0.41	9.60±0.45
				Day 20	9.19	7.27	8.35±0.32	7.04±0.15	15.03±0.92	10.61±0.41
				Day 30	9.29	5.75	6.56±0.21	6.35±0.15	11.14±0.76	7.01±0.60
				Day 40	10.90	6.65	6.57±0.12	6.70±0.18	12.52±0.40	6.86±0.40
	Genetic resource	YCPL243	9.6	Day 10	9.16	6.81	7.10±0.15	7.59±0.80	15.44±0.21	9.75±0.50
				Day 20	9.26	7.00	8.02±0.11	7.77±0.29	16.03±0.18	10.26±0.24
				Day 30	10.60	4.57	8.46±0.18	9.67±0.13	15.07±0.53	9.19±0.35
				Day 40	10.16	5.86	8.25±0.16	9.17±0.18	14.89±0.70	8.78±0.29
Flower	Cultivars	Dayu	9.5	Day 10	9.15	8.58	17.88±1.62	16.63±0.74	34.99±1.14	23.99±1.55
				Day 20	9.25	9.90	20.64±0.47	20.36±0.20	38.30±0.62	35.81±0.50
				Day 30	10.50	8.28	9.95±0.21	8.64±0.23	20.81±0.34	11.79±0.14
				Day 40	10.15	4.20	6.25±0.13	6.01±0.13	12.91±0.51	6.53±0.21
		Anyu	8.25	Day 10	9.40	9.42	24.31±0.22	26.76±0.61	44.78±0.72	31.26±0.13
				Day 20	9.14	10.43	27.91±0.53	31.87±0.20	50.95±0.65	40.20±0.10
				Day 30	9.24	8.21	16.72±0.42	16.21±0.18	29.99±0.40	22.92±0.27
				Day 40	10.40	4.41	6.13±0.21	5.64±0.11	13.67±0.76	5.85±0.22
	Elite Line	Milyang 78	9.2	Day 10	9.12	8.73	20.10±0.43	19.31±0.42	37.70±0.41	26.77±0.44
				Day 20	9.22	10.07	22.04±0.16	22.22±0.19	41.53±0.93	35.80±0.45
				Day 30	10.20	7.81	13.10±0.09	11.06±0.12	25.93±0.72	17.13±0.26
				Day 40	10.12	5.71	6.39±0.12	4.47±0.15	14.30±0.43	6.96±0.15
		Milyang 90	8.3	Day 10	9.90	9.73	26.02±0.32	29.89±0.20	52.47±0.58	32.43±0.46
				Day 20	9.19	13.18	30.64±1.24	33.71±0.26	55.54±0.55	40.57±0.07
				Day 30	9.29	9.01	22.50±1.56	23.14±0.35	40.99±0.64	34.87±1.01
				Day 40	10.90	5.88	8.00±0.31	6.93±0.32	16.09±0.85	7.47±0.35
	Genetic resource	YCPL243	9.6	Day 10	9.16	9.31	17.83±1.68	20.21±0.28	41.02±0.60	22.66±0.32
				Day 20	9.26	11.30	22.57±1.82	21.90±0.05	42.49±0.88	37.89±0.39
				Day 30	10.60	9.08	15.94±1.06	15.19±0.28	30.36±0.92	20.08±0.61
				Day 40	10.16	4.80	7.42±0.42	7.66±0.07	15.06±0.65	7.95±0.28

Supplementary Data S3.

Part	Varieties		Flowering	Growth	Harvest	Phenolic compound content (μg/g)				
			Stage	Periods	Stage	CA[1)]	RA[2)]	RT[3)]	AG[4)]	Total
Seed	Cultivars	Dayu	9.5	Day 10	9.15	149.54±12.88	2146.11±37.46	97.93±2.72	106.64±4.21	2500.21±35.52
				Day 20	9.25	171.13±17.37	2269.23±144.67	101.11±4.98	118.98±6.72	2660.46±140.08
				Day 30	10.50	141.53±3.20	2093.93±102.12	136.32±5.23	141.82±7.48	2513.60±114.37
				Day 40	10.15	97.95±5.73	1743.75±79.13	146.58±6.29	123.62±2.67	2111.91±85.36
		Anyu	8.25	Day 10	9.40	175.30±2.86	2923.89±45.64	121.52±4.36	63.46±0.44	3284.17±48.87
				Day 20	9.14	269.84±10.23	3098.20±80.63	127.60±2.54	68.70±0.47	3564.34±91.60
				Day 30	9.24	212.58±2.56	2850.07±107.73	149.70±9.19	66.81±3.60	3279.16±119.24
				Day 40	10.40	193.09±19.02	2268.02±106.45	156.10±8.92	58.42±0.71	2675.63±116.00
	Elite Line	Milyang 78	9.2	Day 10	9.12	155.39±7.62	2480.35±44.66	498.57±58.88	161.86±7.47	3296.17±102.97
				Day 20	9.22	199.20±10.48	2964.59±59.73	551.66±20.47	174.86±7.79	3890.30±73.59
				Day 30	10.20	181.75±17.70	2724.69±82.75	641.25±26.64	205.81±4.82	3753.49±117.08
				Day 40	10.12	134.83±5.99	2088.48±54.36	650.96±14.04	174.22±7.33	3048.49±60.75
		Milyang 90	8.3	Day 10	9.90	181.06±4.55	2644.62±58.80	478.70±33.58	220.15±1.48	3524.54±90.18
				Day 20	9.19	281.01±11.37	3129.10±138.38	627.67±66.98	234.61±3.42	4272.39±126.33
				Day 30	9.29	233.55±1.98	2885.90±45.55	703.58±28.78	253.86±5.41	4076.89±65.62
				Day 40	10.90	159.48±9.20	2512.43±101.81	783.89±11.98	243.01±3.42	3698.82±103.81
	Genetic resource	YCPL243	9.6	Day 10	9.16	105.57±7.57	2588.05±40.23	222.44±12.96	112.68±5.01	3028.74±23.21
				Day 20	9.26	167.01±4.82	2920.77±34.52	256.01±4.03	125.61±4.71	3469.42±34.25
				Day 30	10.60	137.87±2.64	2836.64±46.34	298.6±10.74	147.27±2.97	3420.38±54.78
				Day 40	10.16	104.34±2.80	2039.67±61.50	314.48±8.50	131.22±7.26	2589.72±56.23
Flower	Cultivars	Dayu	9.5	Day 10	9.15	269.96±4.87	2433.65±47.10	174.48±6.03	173.53±4.01	3051.62±48.45
				Day 20	9.25	305.09±9.84	2956.27±32.38	195.13±13.34	185.31±6.21	3641.80±51.08
				Day 30	10.50	243.99±9.47	2823.76±105.31	220.97±9.51	209.63±2.19	3498.35±92.74
				Day 40	10.15	198.23±12.54	2028.45±42.48	256.33±5.66	193.77±11.31	2676.77±38.48
		Anyu	8.25	Day 10	9.40	366.71±12.01	3432.75±94.38	172.21±6.67	112.85±3.99	4084.52±92.36
				Day 20	9.14	408.19±12.00	3805.16±57.46	169.31±7.60	129.07±19.13	4511.74±64.77
				Day 30	9.24	340.05±1.12	3485.27±127.48	176.37±4.61	154.59±4.53	4156.28±124.84
				Day 40	10.40	240.28±7.04	2667.01±207.62	218.81±7.69	135.55±1.18	3261.64±211.48
	Elite Line	Milyang 78	9.2	Day 10	9.12	283.34±5.34	2714.22±71.93	600.71±3.90	297.74±6.78	3896.00±66.58
				Day 20	9.22	353.75±11.37	3245.61±51.18	674.82±11.86	324.64±13.67	4598.82±59.11
				Day 30	10.20	313.82±7.82	2957.58±40.12	744.30±26.27	367.57±6.27	4383.27±55.19
				Day 40	10.12	223.17±10.58	2178.71±49.80	753.68±16.88	325.61±10.75	3481.17±17.31
		Milyang 90	8.3	Day 10	9.90	376.5±5.01	3125.45±86.31	769.71±2.73	482.32±9.94	4753.97±81.83
				Day 20	9.19	421.72±7.26	3930.41±64.01	837.66±25.05	510.74±25.87	5700.53±121.29
				Day 30	9.29	337.23±10.99	3452.76±135.29	931.45±58.69	533.40±63.39	5254.85±132.10
				Day 40	10.90	235.11±1.30	2807.33±91.25	980.68±55.64	482.01±10.21	4505.12±153.33
	Genetic resource	YCPL243	9.6	Day 10	9.16	233.47±5.43	2576.26±81.43	397.50±10.48	232.28±7.08	3439.51±92.51
				Day 20	9.26	329.58±6.26	3358.16±71.38	413.46±26.78	248.18±2.32	4349.38±51.84
				Day 30	10.60	268.77±29.88	3166.57±82.34	477.68±33.81	270.48±19.05	4183.50±61.27
				Day 40	10.16	270.38±2.69	2160.48±61.51	512.18±40.55	238.69±8.48	3181.73±18.61

Supplementary Data S4.

Part	Varieties		Flowering Stage	Growth Periods	Harvest Stage	Polycosanol content (μg/g) HC[1)]	OC[2)]	TC[3)]	Total[4)]
Seed	Cultivars	Dayu	9.5	Day 10	9.15	26.90±0.23	47.28±1.25	57.51±1.38	131.69±2.84
				Day 20	9.25	34.99±0.55	66.69±2.68	61.24±0.68	162.92±3.87
				Day 30	10.50	47.56±0.09	83.40±0.65	54.09±0.12	185.05±0.62
				Day 40	10.15	54.30±0.39	82.11±1.35	39.60±1.81	176.01±2.76
		Anyu	8.25	Day 10	9.40	24.17±0.25	41.28±0.64	55.70±0.95	121.16±1.17
				Day 20	9.14	30.76±0.75	53.85±0.87	52.64±0.87	137.24±0.43
				Day 30	9.24	43.30±0.00	75.29±0.07	48.42±0.35	167.01±0.41
				Day 40	10.40	52.22±2.01	70.89±0.55	35.92±0.97	159.03±2.54
	Elite Line	Milyang 78	9.2	Day 10	9.12	29.22±0.53	50.98±0.75	58.06±1.21	138.25±1.42
				Day 20	9.22	37.23±0.70	71.79±0.99	55.28±0.34	164.30±0.82
				Day 30	10.20	48.31±0.09	90.29±0.40	50.21±0.34	188.82±0.84
				Day 40	10.12	59.58±3.42	80.47±2.73	40.11±0.46	180.16±5.09
		Milyang 90	8.3	Day 10	9.90	27.26±0.42	49.25±1.28	58.36±1.18	134.87±1.45
				Day 20	9.19	34.52±0.72	66.03±0.61	59.68±0.86	160.24±1.03
				Day 30	9.29	47.73±0.05	85.43±0.50	49.20±0.40	182.36±0.84
				Day 40	10.90	51.45±1.36	81.48±0.90	43.18±1.44	176.10±0.89
	Genetic resource	YCPL243	9.6	Day 10	9.16	24.21±0.45	43.44±0.84	56.49±2.07	124.14±1.31
				Day 20	9.26	30.18±0.71	62.27±1.02	51.08±2.30	143.54±1.58
				Day 30	10.60	45.02±0.08	82.95±0.60	48.50±0.20	176.47±0.63
				Day 40	10.16	48.04±1.07	79.94±0.55	36.97±1.86	164.95±3.19
Flower	Cultivars	Dayu	9.5	Day 10	9.15	21.31±0.36	32.86±0.76	40.56±1.36	94.73±0.50
				Day 20	9.25	27.45±0.24	43.09±0.64	38.37±0.64	108.92±0.21
				Day 30	10.50	36.08±1.36	53.52±0.59	29.13±1.85	118.73±0.61
				Day 40	10.15	41.22±0.74	49.35±1.32	22.11±0.42	112.69±1.63
		Anyu	8.25	Day 10	9.40	18.50±0.20	26.14±0.14	32.80±2.06	77.43±1.72
				Day 20	9.14	24.05±0.35	32.44±0.47	33.58±1.27	90.07±1.06
				Day 30	9.24	33.10±0.67	43.76±0.69	22.58±0.29	99.44±1.42
				Day 40	10.40	38.00±0.55	41.00±0.23	18.50±0.27	97.50±1.04
	Elite Line	Milyang 78	9.2	Day 10	9.12	22.73±0.46	34.85±0.34	44.20±0.97	101.78±1.60
				Day 20	9.22	29.04±0.59	48.01±0.88	40.62±1.34	117.68±0.90
				Day 30	10.20	38.82±0.89	61.62±1.96	30.75±1.34	131.19±2.92
				Day 40	10.12	47.30±1.14	57.37±0.92	24.25±0.52	128.92±0.65
		Milyang 90	8.3	Day 10	9.90	21.68±0.30	33.59±0.51	42.73±0.80	98.00±0.85
				Day 20	9.19	30.44±0.29	45.46±0.89	37.61±0.62	113.51±1.75
				Day 30	9.29	41.42±1.10	58.02±0.76	36.20±1.62	135.64±2.83
				Day 40	10.90	47.57±1.00	55.93±0.57	22.95±0.66	126.44±1.48
	Genetic resource	YCPL243	9.6	Day 10	9.16	18.79±0.15	25.91±0.05	32.52±0.53	77.21±0.44
				Day 20	9.26	24.43±0.36	31.42±0.37	31.06±1.32	86.91±1.37
				Day 30	10.60	34.07±0.42	40.12±0.71	21.55±0.51	95.74±0.75
				Day 40	10.16	37.80±0.27	40.37±0.42	17.73±0.77	95.90±0.63

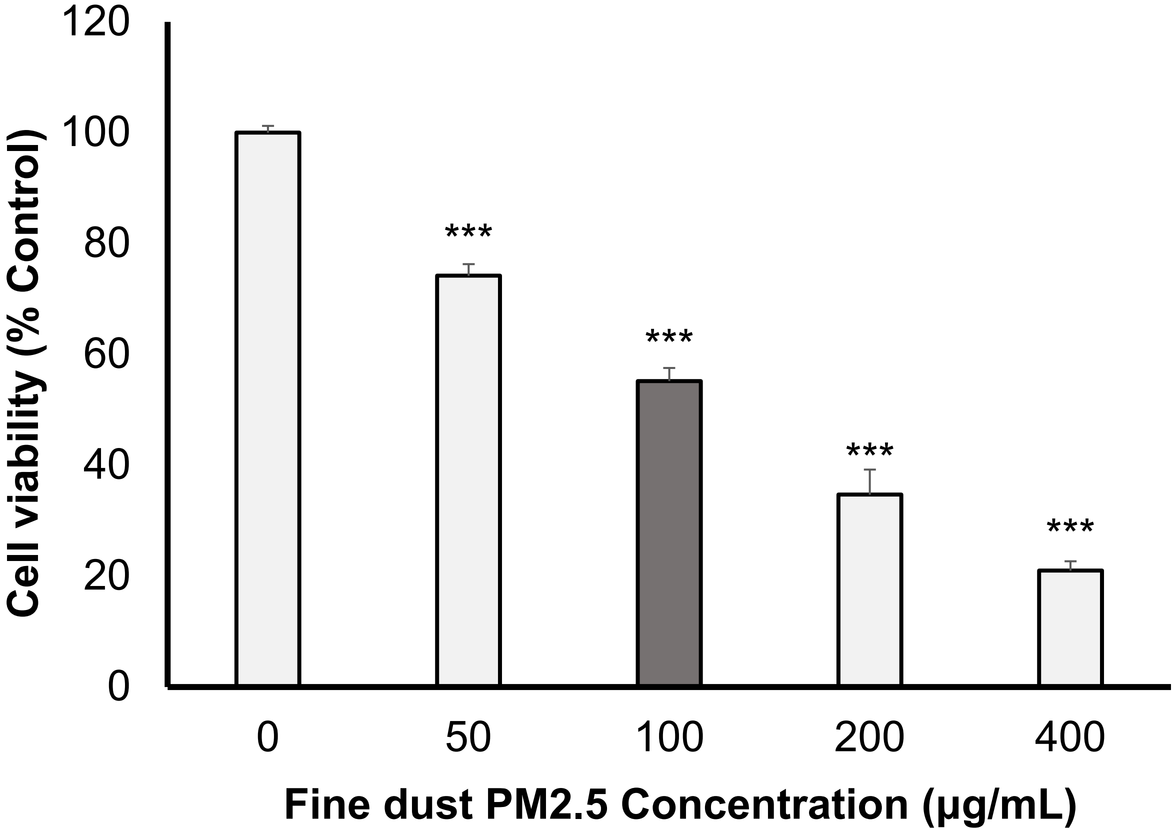

Supplementary Data S5.
